# Editorial: Understanding how the physiology of insect vectors influences vector-borne disease transmission

**DOI:** 10.3389/fphys.2023.1259802

**Published:** 2023-07-27

**Authors:** Katia C. Gondim, Natraj Krishnan, Petros T. Damos, Amr A. Mohamed, Mohammad Mehrabadi, Maria L. Simões

**Affiliations:** ^1^ Instituto de Bioquímica Médica Leopoldo de Meis, Universidade Federal do Rio de Janeiro, Rio de Janeiro, Brazil; ^2^ Department of Biochemistry and Molecular Biology, Entomology and Plant Pathology, Mississippi State University, Starkville, MS, United States; ^3^ Department of Agriculture, School of Agricultural Sciences, University of Western Macedonia, Florina, Greece; ^4^ Department of Entomology, Faculty of Science, Cairo University, Giza, Egypt; ^5^ Department of Entomology, Faculty of Agriculture, Tarbiat Modares University, Tehran, Iran; ^6^ Department of Biomedical Sciences, Institute of Tropical Medicine Antwerp, Antwerp, Belgium

**Keywords:** insect vector, physiology, *Rhodnius prolixus*, *Phlebotomus papatasi*, *Anopheles stephensi*

## Introduction

The field of medical entomology is becoming more relevant globally, with the emergence or resurgence of vector-borne diseases. The rate and efficiency of insect-vectored infectious diseases transmission relates to the physiology of the insect vector, and the interactions insects establish with the pathogens they transmit, at the tissue, cellular and molecular levels. The present Research Topic *Understanding how the physiology of insect vectors influences vector-borne disease transmission* highlights the importance of deciphering physiological mechanisms that influence disease transmission in medically relevant insect vectors.

## Rhodnius prolixus

*Rhodnius prolixus*, commonly known as the “kissing bug,” is a significant vector in the transmission of Chagas disease in Latin America (de Fuentes-Vicente et al., 2018). This disease affects 6-7 million people worldwide (WHO, 2023a) and is caused by the protozoan parasite *Trypanosoma cruzi*. *R. prolixus* egg production is triggered by the ingestion of blood by the adult female. Nutrients and energy needed for embryo development are stored in the form of yolk. After fertilization, the yolk granules play a role in controlled mechanisms to break down the yolk, providing substrates for the growing embryo (Ramos et al., 2022). However, the specific mechanisms involved in the regulated mobilization of yolk are still not well understood. De Almeida et al. investigated the role of maternally accumulated mRNAs in the degradation of yolk and reproduction in *R. prolixus.* They focused on a protein phosphatase, PP501, and two aspartic proteases, cathepsin-D 405 and cathepsin-D 352. They observed that PP501 and CD352 were highly expressed in the *R. prolixus* ovaries undergoing vitellogenesis. Silencing PP501 through RNA interference (RNAi) led to a significant decrease in oviposition and increased embryo mortality, while silencing CD352 resulted in a minor decrease in oviposition and embryo viability. To understand the reproductive impairment caused by PP501, the authors examined the biogenesis of yolk granules during oocyte maturation and discovered that yolk granule formation was impaired during oogenesis. Furthermore, while the fertilization of PP501-silenced eggs occurred, a disruption in yolk granule acidification and acid phosphatase activity led to a complete failure in the degradation of yolk proteins. Based on these findings, the authors concluded that PP501 is essential for oocyte maturation and the activation of yolk degradation, thereby playing a crucial role in *R. prolixus*’ reproduction.

Lipid metabolism, another important aspect of insect physiology, is critical for growth and reproduction and for energy demands during prolonged nonfeeding periods. De Paula et al. investigated the role of carnitine palmitoyltransferase I (CPT1), the enzyme that converts long-chain acyl-CoA species to their corresponding long-chain acyl-carnitines in the outer mitochondrial membrane for transport into the mitochondria, in lipid metabolism and in resistance to starvation in *R. prolixus*. They used qRT-PCR to track the expression of the *RhoprCpt1* gene in the adult female organs 4 days post-blood meal. *RhoprCpt1* was highly expressed in the flight muscle, while dramatically decreased in the fat body after feeding, then increased again 10 days later. No changes were detected in the flight muscle. Further, β-oxidation rates were the highest in the flight muscle, with no changes in the fat body, even with the presence of etomoxir or malonyl-CoA. Silencing of *RhoprCpt1* through RNAi resulted in increased amounts of triacylglycerol and larger lipid droplets in the fat body of females but not in the flight muscle. RhoprCPT1-deficient females had a shorter lifespan after the induced starvation. This reveals the key role of CPT1 in lipid mobilization, where the inhibition of *RhoprCpt1* expression negatively affected lipid mobilization while decreasing resistance to starvation. These findings constitute a significant piece of information that was required for establishing environmentally friendly control practices for this blood sucking insect.

Chitin serves as a structural polysaccharide, providing strength and rigidity to the exoskeletons of arthropods, and chitinases are enzymes that break chitin down into smaller oligosaccharides or monomers (Merzendorfer and Zimoch, 2003). In their paper, Gama et al. focused on the annotation and characterization of chitinase and chitinase-like protein genes in the genome of *R. prolixus* through RNAi-mediated silencing. Knockdown of the *RpCht7* gene resulted in increased mortality in starving nymphs and reduced female oviposition, since silencing *RpCht7* also led to a reduction in the number of eggs laid by female insects. However, it did not affect other physiological parameters such as blood intake, digestion, or molting. These findings suggest that RpCht7 plays a role in the reproductive physiology and vector fitness of *R. prolixus*. Identifying the key role of chitinase genes that are essential for reproduction or other key aspects of vector biology can offer potential targets for the development of novel vector control strategies for *R. prolixus*.

## Phlebotomus papatasi

*Phlebotomus papatasi* is an important vector of *Leishmania major*, an etiological agent of cutaneous leihmaniasis (Akhoundi et al., 2016), of which 600,000 to 1 million new cases are estimated to occur worldwide annually (WHO, 2023b). Understanding how the sand fly reacts to the *Leishmania* infection may unravel potential targets for decreasing transmission. Antimicrobial peptides (AMPs) act to control infectious agents. In insects, AMPs’ transcription is mainly regulated by the Toll and immune deficiency (IMD) pathways. In *P. papatasi*, the gene expression of a previously identified gut-specific AMP, *PpDef1*, was shown to increase after *L. major* infection (Kykalová et al., 2021). Kykalová et al. then investigated whether this response was regulated by the IMD pathway, and if defensins have a role in the control of parasites. A second identified defensin, PpDef2, was silenced by RNAi-mediated gene silencing. Knocking-down PpDef1 or both PpDef1 and PpDef2, resulted in increased parasite levels and higher sand fly mortality. Interestingly, the knockdown of relish in the insect carcass reduced the gene expression of *PpDef2* and attacin, another AMP. These results indicate the central role of defensins in sand fly response toward *L. major* and the importance of the IMD pathway in regulating AMPs in *P. papatasi*.

## Anopheles stephensi

*Anopheles stephensi* is one of the most important malaria vectors. Historically a South Asian mosquito, it has been expanding to the African continent during the last decade. Over 619,000 deaths and 247 million cases of malaria were registered globally in 2021 (WHO, 2022). Individuals with severe *Plasmodium falciparum* malaria (the deadliest malaria parasite) often exhibit reduced levels of 5-hydroxytryptamine (5-HT), and these changes are associated with disease pathology. In insects, 5-HT is a neuromodulator that regulates life history traits and behavior. 5-HT therefore varies in concentration in human blood with malaria and can signal to the mosquito following blood feeding. Briggs et al. investigated the impact of ingested 5-HT in the *An. stephensi* physiology. They provisioned 5- HT at normal blood levels (1.5 µM) and at levels associated with severe malaria (0.15 µM). Ingested 5-HT had no impact on *An. stephensi* oviposition, lifespan nor feeding behavior. Mosquitoes provisioned with 5-HT associated with severe malaria exhibited significantly increased flight velocity in response to visual and olfactory cues. 5-HT ingested in blood enhanced the tendency of uninfected mosquitoes to take a second blood meal after 4 days following the first one. Importantly, treatment of *An. stephensi* with 5-HT associated with severe malaria increased infection success with mouse *P. yoelii,* while treatment with healthy levels reduced infection success with human *P. falciparum*. In summary, the authors demonstrated in their work how 5-HT influences mosquito physiology and behavior.

In conclusion, by focusing on the physiological aspects of insect vectors, this Research Topic provides valuable insights that can inform the development of effective strategies to control some of the most important insect vectors and the diseases they transmit.
